# An Epitome of Some Questions of the Day

**Published:** 1909-12-25

**Authors:** 


### THE YEAR'S PROBLEMS.

### AN EPITOME OF SOME QUESTIONS OF THE DAY.

There are certain questions daily growing in importance which no professional man in this country can afford to disregard. To members of the medical profession in particular these questions, some of which involve the very issues of our national existence, should be of absorbing interest. They are the men and women who can take an intelligent concern in matters which demand an acquaintance with scientific data such as the average layman does not possess. They, therefore, are the men and women to whom the public look, not unnaturally, for enlightenment and for information upon sociological and semi-scientific problems, questions of the day which are being daily discussed, matters upon which the opinion of every practitioner may be asked by his patients. The general practitioner is a busy individual and cannot be expected to wade through blue books and special reports, and it has appeared to us that at this season of the year there may be many in the profession who would appreciate a brief epitome or summary of the more important matters which have been touched upon during the year in both the lay and the professional press. With this object in view we have compiled the following notes, which may serve to keep our readers in touch with some of these problems. It is unnecessary to state that in many cases political feelings enter largely into the discussion of several of these items; with such we cannot deal, but there remain a number of serious questions which have arisen during the year. With these every medical man should be familiar enough to be able to express an opinion upon them. The medical profession is gradually becoming alive to the duties and responsibilities which are imposed upon all its members in virtue of their professional status and the confidence which is placed upon them. So many important questions of the day concern the profession as a whole, into the discussion of so many others again scientific considerations enter largely, and the influence which practitioners as a class can exercise in educating the community is so great that such questions ought to be fully understood by every medical man. No apology, then, is needed for these brief summaries. They are elementary, and those who are interested in the subjects with which they deal should endeavour to become thoroughly familiar with them in all their various aspects and should study them from all points of view.

### The Position of Women.

So far as the medical profession is concerned the position of women has been definitely settled by the action of the Royal Colleges, which this year have granted women students practically equivalent facilities to those already possessed by male students. With the exception of the Universities of Oxford and Cambridge, which still refuse to recognise the professional equality of the two sexes, there are no licensing bodies in the United Kingdom which exclude women from their ranks. It is true there are still many stumbling-blocks in the way of the woman diplomate occupying the same position as her male competitor. The charter of the Royal College of Surgeons, for example, prevents women diplomates, Members or Fellows, from voting or taking a seat upon the council, serving as examiners, or having any actual share in the management of the college. The action recently taken by the Manchester Infirmary in refusing to allow women graduates to become candidates for resident appointments, again, shows that there are practical difficulties in the way of women students. These difficulties will doubtless be lessened in time. During the past year women graduates have shown their capabilities in many directions, chiefly, perhaps, as medical inspectors of school children. With the political questions at issue we have no desire to deal beyond saying that the questions of woman's suffrage and adult suffrage are likely to attract considerable attention during the ensuing year, and that it behoves medical practitioners to form an opinion upon them.

### Professional Problems.

During the past year many points have arisen which should have the consideration of practitioners who have at heart the interests of the profession. With these we have dealt, as they [illegible]rose, in The Hospital, and it is sufficient to al[illegible]de very briefly here to some of the more important ones. Easily first comes the question of medical education, which has been discussed very fully by the General Medical Council. The agitation to modify the curriculum has so far produced little result. It has been generally accepted that as matters stand the curriculum is unsatisfactory: the five years' minimum, designed originally to allow the student to become more fully proficient in practical clinical work, is being wasted, in the majority of cases, in preliminary work. Scientific requirements are gradually increasing, and there is ground for the feeling that too little time is given the student to enable him to work at his final subjects, with the result that the length of the course, in the average, has been raised to six and a half years. The additional expense involved by this has been generally commented upon, but nothing has so far been done to revise the curriculum, beyond allowing the student to do a portion of his preliminary work before registration. Closely connected with this question of medical education in general is the position of the diplomate, and the London diplomate in particular. It has been alleged that in consequence of the severity of the preliminary work required by the medical examinations of the London University—especially the ridiculous and unequal standard maintained in spite of all protests by its matriculation examination—the percentage of students taking the London degrees is decreasing, and that students are flocking to provincial centres, which attract them owing to the facilities they offer for medical graduation. As a consequence of this attitude on the part of the metropolitan university, the London schools are finding it increasingly difficult to get an average entry, while the vast majority of students who study at these schools are handicapped in after life by the fact that they are diplomates and not graduates, unable to lay legal claim to the title of "Doctor," and, unless they have taken the higher diplomas which involve far more work than the average provincial degree, debarred from seeking election on a hospital staff, or applying for higher appointments. There has, therefore, been an attempt to obtain for London diplomates greater facilities to graduate—so far unsuccessfully. The various licensing bodies and medical schools have expressed themselves in favour of such facilities, and negotiations are at present proceeding with the object of enabling their diplomates to win for themselves the right to style themselves Dr. Whether these attempts will result in anything practical being accomplished remains to be seen. The senate of the London University is naturally averse to lowering its degree standard, and no legislative action is likely to be taken in the near future with a view to introducing something akin to the "State examination" such as prevails in Continental countries. Those who are interested in the subject should read Dr. Elliot-Blake's exposition in "Medical Reform Measures." (Bale, Sons and Danielsson, London.)

### The Nationalisation of Medicine.

In this matter, which has been touched upon spasmodically during the past year, little progress has been made. An association of medical men in favour of what may be called "socialistic reforms" in the profession, has been formed, and has already gained many adherents. Such matters as the medical inspection of school children, the establishment of school clinics, and the social-hygienic reforms which have been accomplished during the year, have drawn attention to the programme of this association, but it is too early yet to say how far the profession as a whole is interested in the matter. Considerable attention has been paid to the parliamentary representation of the profession, and it is interesting to notice that in the forthcoming election a greater number of medical men are soliciting the suffrages of the electorate than in any previous election. The parliamentary work in which medical men have proved their value as legislators during the year has been important. It has included such measures as the Asylums Officers' Superannuation Act, the Cinematograph Act, and the Housing Act, while a great deal of excellent work has been done in committees.

### Medical Research.

Gratifying progress has been made in this important subject, especially in cancer investigations and research in tropical medicine and tuberculosis. The Sleeping Sickness Bureau has done important work, while the activities of special cancer research scholars at the Middlesex and elsewhere have been most satisfactory. Equally gratifying is it to record the liberal support that medical research has gained during the past year in the shape of endowments and grants. Many hospitals and medical schools have been enabled to endow special scholarships for research purposes owing to the munificent liberality of supporters. In particular mention must be made of the late Mr. Barnato's large bequest which has been handed over to the Middlesex Hospital for the establishment of special cancer wards, and of Mr. Otto Beit's donation of £210,000 to the London University for the endowment of Beit Fellowships in special research. In tropical medicine the Liverpool School has benefited largely through the liberality of the late Sir Alfred Jones and other friends, while the British Medical Association and the Royal Society have both taken their usual share in stimulating research in various departments.

### Anæsthetics.

A feature of the year has been the attention paid to anæsthetics, largely in consequence of the undue amount of interest displayed by the public in the fatalities which have attended the administration of general anæsthetics. During the year no less than 209 such fatalities have been recorded, the large majority due to chloroform, and coroners in various parts of the kingdom have thought it their duty to comment upon these cases. An attempt has been made to interest the Legislature in the subject, and a bill has been drafted providing that no general anæsthetic should be administered except by a qualified medical man, and that every medical student, before obtaining his qualification, should prove that he has had a satisfactory education in the administration of anæsthetics. This bill, however, has not met with the approval of the profession, and it is obvious that its provisions are crude and unsatisfactory; but the general publicity given to the matter has done good in so far that it has drawn attention to the whole subject of anæsthetics, and showed how eminently desirable it is that students should pay more attention to this branch of medical study than they have hitherto done. Amended regulations providing for such extra instruction in the administration of general anæsthetics have been enforced in many schools, with the eminently gratifying result that the standard of efficiency has been raised. At the same time it is generally felt that the time has arrived when the whole subject should be reviewed, and there appears to be a desirability that a Governmental Commission should be appointed to investigate the matter with a view of reporting on the advisability of taking legislative action.

### Vivisection.

The Royal Commission empowered to investigate into this subject is understood to have finished its labours, but it has as yet issued no final report, although several interim reports containing the evidence given before it have been published from time to time. No explanation of the delay involved in preparing the final report has been given, and it is impossible to say when it will be issued. The anti-vivisection agitation has been very active during the past year, and has been specially to the front in connection with hospital matters. As an offset against it, the Research Defence Society has been vigorously active and has done much to educate the public. It has held many meetings and distributed a mass of literature showing the benefits which have been derived from research, and pointing out how progress in treatment has gone hand in hand with investigation into the ætiology and pathology of disease.

### "Christian Science" and Quackery.

Attention must be drawn to the propaganda of Mrs. Eddy's followers, whose methods have been exposed on more than one occasion during the past year. Mr. Stephen Paget has identified himself closely with the work of exposure, and his addresses to the Church Congress and other bodies deserve to be read by every practitioner who desires to take an intelligent interest in the subject. The subject of quackery in general has come in for more than ordinary notice during the year, for one reason owing to certain actions at law, and for another owing to the detailed accounts of many quack schemes which have appeared in the Press. Dr. Walsh has done good service in directing the attention of the profession to the matter in a series of articles which appeared in the *Medical Press and Circular* and have since been re-published in pamphlet form; while the concise exposures contained in "Secret Remedies," published by the British Medical Association, should prove helpful to every practitioner in discussing the subject. In Australia and Germany the year has seen legislative action in regard to such matters, and it is possible that before very long similar legislative action for the protection of the public against manifestly fraudulent and barefaced impostures may have to be taken in this country.

### The Year's Work in Parliament.

We have already alluded to some of the Acts passed during the last session in which medical members of Parliament have demonstrated their active interest. Though this session cannot be held to have been in any way epoch-making so far as the profession is concerned, yet certain measures which have been discussed demand some attention. The Housing and Town Planning Act is an admirable measure which should have the cordial endorsement of the profession—as it has had. The Assurance Act will interest medical men who are occupied in assurance work, while Sir William Collins's two Acts, the Metropolitan Ambulances and the Asylums Officers Superannuation, with the Irish Health Resorts and Watering Places Acts will appeal more especially to certain sections of the profession—those interested in institutional and public health questions. Sir William Collins has won the distinction of passing two out of a total of eight private Acts last session. The Short Oaths Act is of more general interest, since it provides for an every-day occurrence in a manner that will be approved by every medical witness. The Local Education Authorities (Medical Treatment) Act is a step in the right direction, since it provides the local authorities with the means of recovering the cost of medical treatment for school children in certain instances. Several useful measures, unfortunately, have been slaughtered, mainly owing to want of time for their adequate discussion. Among these are the Indecent Advertisements Bill, certain Bills with regard to public health, drainage, and food supply, and some interesting private Bills. Among the contentious measures introduced, but hardly discussed, were the Nurses Registration Bill, a Bill exempting dogs from being used for vivisection purposes, the Coroners Inquest Bill, and the Daylight Saving Bill. Much useful information was made public in the official replies to questions, many of them not answered orally. In the debates on the estimates, in particular, on the Home Office, War Office, and Local Government Board votes, many interesting questions were touched upon and dealt with.

### Post Graduate Study.

The year has seen an important endeavour to internationalise graduate study in medicine. Mainly owing to the energy of Professor R. Kutner, of the Kaiserin Friedrich Haus, Berlin, who has interested himself greatly in the movement, a special committee was formed at the recent International Medical Congress held at Budapest with the object of promoting the interests of medical graduate students. The committee is a strong and representative one, and has already actively engaged in propaganda work. In England and America gratifying progress is being made in graduate study, which, it is satisfactory to notice, is yearly attracting increasing numbers of medical men. Much more energy and a greater general interest are wanted, however, to make the movement really a success in this country, where the facilities for such study are not equal to those offered in America or on the Continent. If public attention would now divert itself from its somewhat excessive preoccupation with medical research—which is now, if anything, over-provided for—and would attempt the endowment of post-graduate study, it would be well for the public and the practitioner. It is to be feared that at the present time the public has not appreciated the importance, even from a selfish point of view, of such secondary medical education. We therefore throw out a suggestion to the profession to popularise this question in the same successful way as has been done for medical research.

### The Medical Inspection of School Children.

This highly necessary reform has been much discussed during the past year, more especially in connection with the attitude of the metropolitan hospitals on the question of the medical treatment of school children. After some debate, the Education Committee of the London County Council, specially authorised to report upon the whole subject, recommended the acceptance of the offer of several London hospitals to treat such children as the medical inspectors certified to be in want of treatment on condition that specific grants were made by the Council to the hospital funds. This has been tentatively agreed upon, and at least five large metropolitan hospitals are now treating such children. The action taken by the Council has not been generally approved by the profession. It is felt that the arrangement entirely ignores the general practitioner, and gives the children over to clinical assistants who may not have had much experience, while at the same time it complicates the question of hospital management owing to the introduction of contract work. As an alternative, the founding of special school clinics has been suggested, but this has not been favourably received. For the present the new arrangement will be given a year's trial, at the end of which period it is probable that important changes and modifications may have to be made. So far the systematic inspection of school children has done good, although there is a tendency to regard as pathological conditions which are not in accordance with the normal average. This tendency is to be deplored, since it gives rise to a totally erroneous impression with regard to the physical deterioration in secondary schools.

The Poor Law and the Feeble-Minded.

Two important reports, with which we cannot deal in full on this occasion, demand the attention of the practitioner who desires to be up to date in discussing the problems, sociological and economical, of the day. These are the report of the Royal Commission on the Poor Law and that of the Commission on the Care of the Feeble-minded. Both reports deal with very important national subjects, and will well repay the close study which a proper understanding of their recommendations involves. The Commission on the Poor Law acknowledges that the present system of outdoor relief is out of date, inefficient, expensive, uneconomic, and wasteful, and that it is imperatively necessary that the chaotic multiplication of statutes dealing with the problem of poverty in its various aspects should be stopped, and an attempt made to codify existing legislative enactments and attack the problem seriously. It is only when the subject of actual reform and the ways and means whereby this much-needed reform is to be accomplished are touched upon that the Commissioners show themselves in total disagreement with one another. The Majority report is wholly different from the Minority suggestions. We cannot here go into these suggestions; suffice it to say that the Minority report proposes to deal with pauper relief in a thoroughly radical—almost revolutionary—fashion, and that one of its suggestions is the appointment of an official registrar. Both reports have been eagerly and acrimoniously discussed, but the subject is so involved, and the mass of evidence so huge and so conflicting, that no one who has read the two reports can plunge lightly into such a debate. It is, however, one of those subjects upon which the opinion of the general practitioner conversant with the essentials of the Poor Law system will be valuable, and for that reason we commend the bulky report to the attention of the profession.

